# Recurrent intestinal intussusception in an adult due to intestinal pseudopolyps not associated with inflammatory bowel disease: a case report

**DOI:** 10.1186/s13256-015-0754-x

**Published:** 2015-11-23

**Authors:** Fernando Martínez-Ubieto, Teresa Jiménez-Bernadó, Alvaro Bueno-Delgado, Javier Martínez-Ubieto, Ana Pascual-Bellosta

**Affiliations:** 1 Digestive and Bariatric Surgery Department, Viamed MontecanalHospital, 50008 Zaragoza, Spain; 2 Service of Anesthesiology, Viamed Montecanal Hospital, 50008 Zaragoza, Spain; 3 Department of Health Sciences, University of Zaragoza, Zaragoza, Spain

**Keywords:** Jejunum, Intussusception, Pseudopolyps, Three-dimensional laparoscopy

## Abstract

**Introduction:**

Intestinal intussusception is very rare in adults and, unlike in children, it is due to an organic cause, mainly benign or malignant tumors, in 90 % of cases. Recurrent intussusception in an adult is even more exceptional, and in the case reported it was due to repeated occurrence of intestinal pseudopolyps, which is exceptional according to the literature. Preoperative diagnosis is difficult, and surgery is always indicated because a tumor is usually present. The surgical procedure may be controversial, as some would prefer desintussusception before resection, while others would advocate initial resection because of the risk of dissemination if a malignant lesion exists.

**Case presentation:**

We report the case of a 34-year-old Caucasian man who underwent emergency laparoscopic surgery for intestinal obstruction and was found to have a jejunal intussusception. Polyps or pseudopolyps, some of them large and causing the intussusception, were seen in the surgical specimen. Our patient had also undergone surgery for intussusception 10 years before, after which the pathological report also noted the presence of these formations.

**Conclusions:**

Recurrent intussusception in adults due to the presence of intestinal pseudopolyps is exceptional and, to the best of our knowledge, this is the first such case reported.

## Introduction

Intestinal intussusception in adults is very rare, and only accounts for approximately 1 % of cases. Unlike in children, in whom intussusception is of an idiopathic etiology, in adults the condition is due to an organic condition in a majority of cases (more than 90 %). Small bowel intussusception is more commonly due to a benign cause, while the opposite occurs with large bowel intussusception. Giant intestinal pseudopolyps are also uncommon and are usually associated with mucosal changes in inflammatory bowel disease. The lack of association of these pseudopolyps with ulcerative colitis or Crohn’s disease is exceptional. They are also an exceptional cause of recurrent intestinal intussusception, an association which has never been reported in the literature to the best of our knowledge.

Diagnosis of intussusception before surgery is not easy. An abdominal ultrasound examination or a computed tomography (CT) scan may be of help, especially when a typical image of intussusception such as the target sign or target mass is seen.

Surgery is indicated in most cases in adults, and usually involves resection of the affected segment. The laparoscopic approach is not common, but has been shown to be feasible. Desintussusception as a first step is highly controversial in many adults with intussusception because of the risk of dissemination in patients with tumors, and is therefore not routinely recommended.

## Case presentation

A 34-year-old Caucasian man attended the emergency room complaining of abdominal pain, nausea, and vomiting for the past 24 hours. He reported diffuse, severe, noncolicky pain, abdominal distention, and no stools or gases in the past 24 hours. A physical examination disclosed abdominal distention and bloating, with diffuse pain. Bowel sounds were absent. A tender mass was palpated in the left iliac fossa, with abdominal guarding and rigidity.

His vital signs were normal. Laboratory tests in the emergency room revealed a white blood cell (WBC) count of 15,000 cells/mm^3^ with a shift to the left.

Our patient had undergone emergency surgery for intestinal intussusception 10 years before. A 23-cm-long jejunal segment was resected and gross examination revealed polypoid lesions, some of them up to 3 cm in size. A microscopic examination revealed pseudopolyps with no other mucosal changes. During this time, our patient had experienced episodes of abdominal pain and distention that resolved spontaneously or required fluid therapy and absolute diet, and resolved with no need for surgery. An abdominal CT scan and colonoscopy had been performed with no abnormal findings. Colonoscopy ruled out inflammatory bowel disease with colonic involvement. Intestinal magnetic resonance imaging (MRI) performed in one of the episodes, 6 months before the reported surgery, showed images consistent with intussusception.

**Fig. 1:**
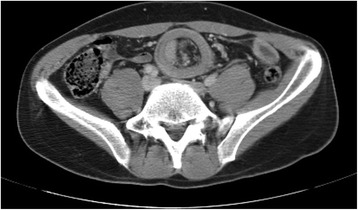
Typical image of "target sign" in the CT scan

An emergency CT scan, performed without oral or intravenous contrast, revealed an image consistent with intestinal intussusception (Fig. 1). Due to persistence of the condition and suspected intestinal intussusception with mesenteric compromise, emergency surgery with a three-dimensional laparoscopic approach was performed. Rocuronium was used as a muscle relaxant. Neuromuscular monitoring was performed with TOF-Watch® SX (Organon Ltd., Dublin, Ireland). Extubation was done when the train-of-four (TOF) ratio was greater than 0.9, and sugammadex 2 mg/kg was administered if the TOF was lower at the end of surgery. Jejunal intussusception was found. Desintussusception was therefore performed, followed by resection of the involved segment. In order to ensure that the ends were not grossly affected by any condition, the intestinal loop was externalized, and extracorporeal resection and anastomosis were performed. The whole small intestine was visualized and was seen to have a grossly normal appearance. The portions of the large bowel visualized also had a normal appearance.

**Fig. 2:**
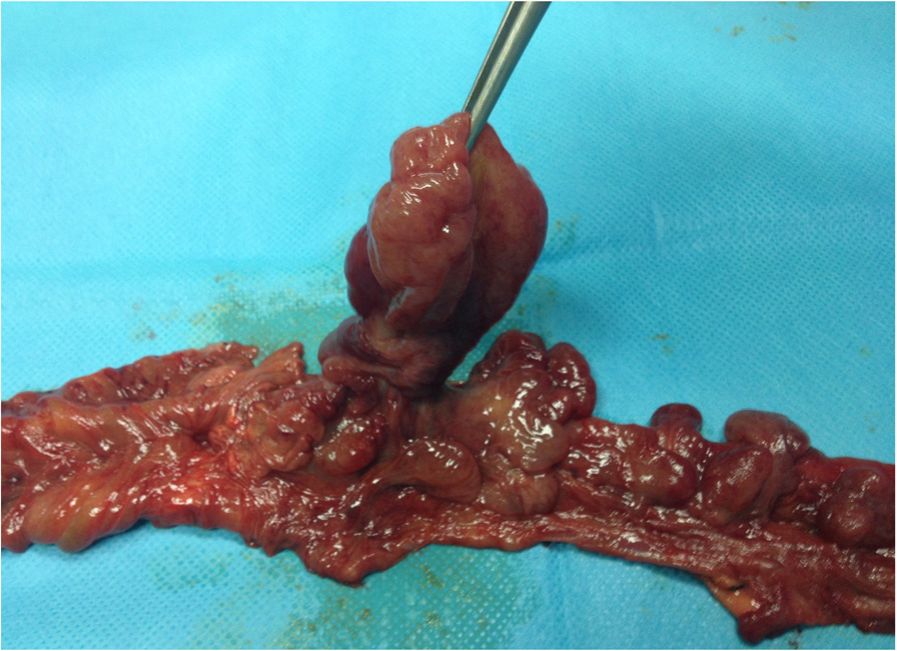
Pathological gross examination with multiple polypoid lesions

Pathological examination found a 20 cm segment of the small intestine, which showed, on gross examination, multiple polypoid lesions of soft consistency not reaching the resection margins (Fig. 2). All polyps present in the surgical specimen were examined. Microscopically, polypoid formations consisted of a core of loose, edematous connective tissue in which the lining epithelium consisted of isolated, slightly tortuous and dilated glands with no atypia. No changes were seen in intestinal mucosa.

The postoperative course was satisfactory and free of complications. Nine months after surgery, a gastrointestinal barium meal examination and colonoscopy were performed, reaching the last centimeters of the terminal ileum, which were normal. Ileum and colon biopsies were normal, showing no signs of inflammatory bowel disease.

## Discussion

There are few reports of intestinal intussusception in adults, and the reported series are small and cover long time periods [1–5]. The most common initial signs include abdominal pain, vomiting, distention and, occasionally, palpable masses. An acute condition often occurs, accounting for 5 % of intestinal occlusions seen at emergency rooms [5]. Intestinal intussusception may sometimes have a subacute or chronic nature, causing intermittent abdominal pain and vomiting. A pathological condition causing the intussusception is found in more than 90 % of the cases. The most common benign lesions in the small bowel include adhesions, Meckel’s diverticulum, submucosal lipoma, and polyps of the Peutz-Jeghers syndrome. Malignant lesions in the small bowel account for 20–50 % of all intussusceptions and are usually due to metastases from tumors such as melanoma, lymphoma, and so on [3]. Malignant lesions are more common in the large bowel, and one of their main causes is colon adenocarcinoma [6]. Small bowel or colonic pseudopolyps are usually associated in the vast majority of cases to inflammatory bowel disease, and may lead to various complications inherent to them, including intussusception [7–9]. Isolated pseudopolyps with no other mucosal pathology are extremely uncommon [10], and intestinal intussusception caused by such pseudopolyps is exceptional.

Clinically, patients with intussusception usually experience sudden abdominal pain associated to nausea and vomiting. Plain X-rays usually show bowel obstruction. Preoperative diagnosis based on suspicion of intussusception is uncommon [3], and abdominal CT is the most helpful test with a high suspicion index when a typical but not pathognomonic image such as the target sign is seen [3, 11]. Ultrasonography may also visualize this same image [12]. 

Most authors agree that surgery should be performed because of the high incidence of organic pathology. The controversial issue is whether the intussusception should be reduced or not. This maneuver may help clearly define the limit of resection, and in specific cases where the cause is an adhesion, the possibility of resecting the affected segment could be dismissed [3, 4, 10]. The problem, however, is that in patients with malignant diseases, manipulation may lead to tumor dissemination by the intraluminal or venous route. Moreover, manipulation favors perforation, and this maneuver should therefore be avoided. However, a preoperative diagnosis of intussusception due to malignant disease is difficult, although concomitant colon intussusception and anemia are by themselves predictors of malignancy, and reduction should therefore be avoided [6]. A laparoscopic approach is possible and may be useful both to reduce intussusception and to perform partial or complete laparoscopic bowel resection [12–14].

## Conclusions

Recurrent intussusception in adults is uncommon, and most often due to an organic cause, unlike in children. Intestinal pseudopolyps are associated to mucosal changes characteristic of inflammatory bowel disease, which did not occur in our case, and we found in the literature no case where pseudopolyps have caused recurrent intestinal intussusception. The laparoscopic approach was possible to resolve this process.

## Consent

Written informed consent was obtained from the patient for publication of this case report and any accompanying images. A copy of the written consent is available for review by the Editor-in-Chief of this journal.
